# Liberation from Non-Invasive Ventilation in Complex Intensive Care Unit Patients

**DOI:** 10.3390/jcm15062148

**Published:** 2026-03-11

**Authors:** Hafsa Safdar, Joseph B. Barney

**Affiliations:** Division of Pulmonary, Critical Care and Allergy Medicine, University of Alabama at Birmingham, Birmingham, AL 35233, USA

**Keywords:** acute hypoxic respiratory failure, hypercapnic respiratory failure, COPD, pulmonary hypertension, morbid obesity, delirium, pulmonary hypertension, palliative care

## Abstract

The evolution of non-invasive mechanical ventilation (NIV) from the iron lung of the 1950s to the use of sophisticated ventilators with mask apparatus has allowed for the optimal management of a wide range of respiratory disorders. NIV is now a mainstay in the management of acute, chronic and acute-on-chronic hypoxemic and hypercapnic respiratory failure from diverse etiologies. While NIV offers an effective approach to avoid invasive mechanical ventilation with its inherent risks of lung injury and sedation-related harms, it is a complex modality that requires a nuanced approach to management As the use of NIV has become ubiquitous, complex challenges are faced in the initiation, management and discontinuation of the treatment. We review complex clinical scenarios that present during liberation from non-invasive mechanical ventilation and an approach to successful weaning and liberation in these patient populations.

## 1. Background and Significance

The polio epidemic of the 1950s highlighted the importance of mechanical ventilatory support for acute respiratory failure and led to the widespread use of the iron lung, a negative-pressure non-invasive ventilator [1]. Bjorn Ibsen saw the high mortality rate for polio-associated respiratory failure and started the practice of positive-pressure ventilation through tracheostomy placement and manual hand bagging, substantially reducing mortality [2]. This emergence of mechanical ventilation is inextricably linked with the creation of the first intensive care units.

Advances in understanding respiratory mechanics and gas exchange therapy for atelectasis and pulmonary edema improved predominantly from the administration of positive end-expiratory pressure (PEEP). Global practice patterns shifted towards positive-pressure mechanical ventilation as well as finding non-invasive approaches to deliver this support without the need for commitment to tracheostomy placement. The mortality and morbidity associated with invasive mechanical ventilation (IMV), including prolonged hospital stays, high sedation requirements, and ventilator-associated pneumonia, contributed to the search for less invasive means of providing short-term ventilatory support. With the recognition of lung protective ventilation and greater understanding of the impact of ventilator dyssynchrony, there was a paradigm shift to provide assistance rather than control of the respiratory cycle, augmenting the patient’s intrinsic respiratory drive and effort. Coupled to this was a growing population of patients with chronic respiratory failure being supported at home, largely with negative-pressure ventilation or positive-pressure ventilation through tracheostomies [1]. A population need was apparent for a non-invasive, more accessible ventilatory modality.

Delaubier and Rideau introduced non-invasive positive-pressure ventilation (NIPPV) for patients with neuromuscular disorders via a nasal mask, and soon this was extrapolated to patients with sleep apnea. In 1992 bilevel positive airway pressure (BIPAP) was introduced to manage patients with sleep apnea who could not tolerate the constant pressure from continuous positive airway pressure (CPAP). This led to increasing understanding of pressure support as a mode of ventilation and the recognition of specific disease processes where it could be used. Randomized controlled trials studying patients with chronic obstructive pulmonary disease (COPD) and congestive heart failure (CHF) showed reduced need for intubation and possible mortality benefits [3,4,5]. The COVID-19 pandemic further solidified the utility of NIV in acute respiratory failure, as thousands of patients were supported with BIPAP without progressing to tracheal intubation. The efficacy of ventilatory support provided via non-invasive mechanisms, growing awareness of potential harms of traditional invasive mechanical ventilation, and the recognition of specific patient populations that benefit from NIV led to its common and daily utilization in the modern intensive care unit (ICU).

Over the last 25 years the use of NIPPV for acute respiratory failure has seen a sharp increase globally. In the United States, multicenter studies show that of all patients requiring mechanical ventilation for acute respiratory failure 38–40% were managed with non-invasive mechanical ventilation [6,7,8].

## 2. Mechanical Effects of NIPPV on Respiration

**Figure 1:**
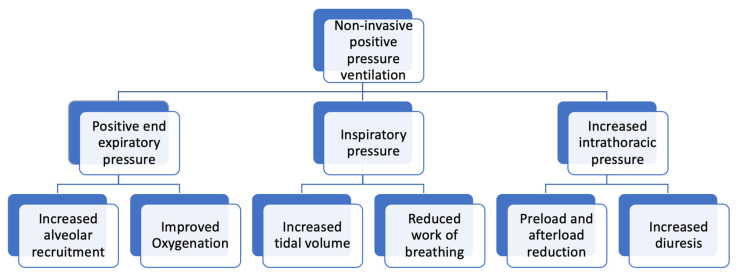
Mechanical effects of non-invasive positive-pressure ventilation.

NIPPV provides an end-expiratory positive airway pressure (EPAP or PEEP) and an inspiratory positive airway pressure (IPAP). Delivery of PEEP improves oxygenation by stenting airways open and preventing end-expiratory airway collapse. This recruitment of collapsed alveoli improves ventilation–perfusion matching and reduces pulmonary shunting, thereby improving oxygenation [9]. The pressure difference between the inspiratory pressure and PEEP functions as a driving pressure. Increases in driving pressure lead to increased tidal volume, in turn improving ventilation and gas exchange. Both effects improve the patient’s work of breathing (Figure 1).

NIPPV also causes an increase in intrathoracic pressure, which leads to reduced preload and afterload. The resultant increase in cardiac output leads to improvement in renal perfusion, increases diuretic effect and improves cardiogenic pulmonary edema.

## 3. Disorders Commonly Managed with Non-Invasive Ventilation

As the use of NIPPV has increased, so has our understanding of its physiologic effects and specific pathologic conditions that derive the most benefit from this treatment modality [10]. It has long been recognized as the optimal treatment modality in acute respiratory failure in COPD exacerbation management [3,4]. Positive-pressure ventilation augments mechanical ventilation by increasing the tidal volume, improves gas exchange and counteracts auto PEEP and dynamic hyperinflation in this patient population.

In patients with cardiogenic pulmonary edema, the use of NIPPV is associated with decreased length of ICU stay and reduced intubation rates. NIPPV improves respiratory mechanics and augments diuresis in these patients [5].

The COVID-19 pandemic saw increased use of non-invasive ventilation in patients with acute hypoxic respiratory failure, with 41% of patients treated with NIV. While data in acute hypoxemic respiratory failure is less clear on its efficacy, if managed correctly, NIPPV can be used successfully as a treatment strategy to prevent intubation in many patients with purely hypoxic lung disease. NIPPV failure rates are much higher in this patient population as compared to those with COPD or cardiogenic pulmonary edema and seem to correlate with severity of hypoxemia. Delayed recognition of NIV failure in patients with moderate to severe acute hypoxemic respiratory failure may be associated with increased mortality.

NIPPV is also increasingly used post-extubation to prevent extubation failure [11]. The key to this approach is identifying high-risk individuals, for example, patients with obesity hypoventilation syndrome, chronic hypercapnia, cardiac disease beyond heart failure and cardiogenic pulmonary edema [12]. Studies have shown reduced reintubation rates and consequently reduced risk of mortality associated with reintubation when the patient population is appropriately selected and NIPPV is initiated [13].

The use of NIV in the post-surgical setting is nuanced. Studies show reduced intubation rates in patients with established post-surgical respiratory failure, especially in high-risk groups like obese patients [14]. One study reported reduced rates of nosocomial pneumonia, ICU length of stay, ICU and hospital stay, as well as long-term mortality [15]. However, prophylactic use of NIV in all-comers post-extubation has not been shown to reduce rates of respiratory failure or intubation rates [16,17]. Patients who are post-operative from gastrointestinal surgeries and at high risk for aerophagia from non-invasive ventilation pose a particular risk for aspiration and should be supported with this modality only in selected cases.

## 4. Complications from Prolonged NIPPV Support

**Figure 2:**
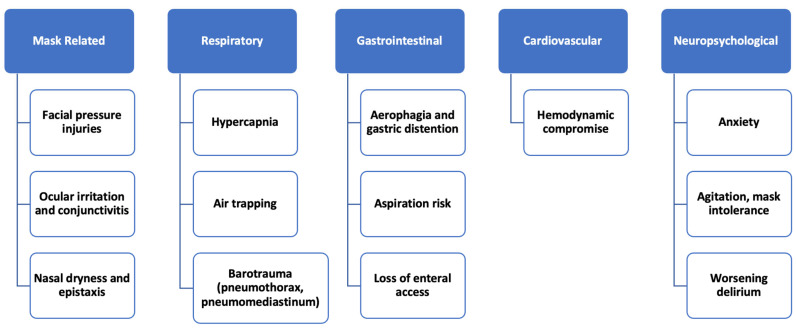
Complications from non-invasive ventilation.

Like any treatment modality NIPPV presents its own unique challenges and complications. Like endotracheal intubation and ventilatory support, the longer it is used continuously, the higher the risk of negative outcomes. The most common complications are outlined below (Figure 2). Face masks are associated with a 20–25% risk of facial pressure injury. The risk of facial decubitus varies with different kinds of face masks [18]. The mainstay of management is prevention by ensuring frequent skin checks, using protective dressings and minimizing pressure [19]. Ocular dryness is caused by air leaks, leading to increased tear evaporation and causing ocular irritation. Using humidified air and ensuring proper mask fit help minimize the effect [20]. Patients receiving positive-pressure ventilation swallow air, leading to gastric distention [21]. This effect seems to be pressure-dependent and is higher with higher airway pressures. A study in intubated patients showed increased risk of ventilator-associated pneumonia in patients with aerophagia and gastric distention [22]. Claustrophobia and anxiety are a common cause of NIPPV intolerance and failure. In a study, 37% of patients on NIV reported anxiety associated with NIV use. Positive-pressure ventilation is associated with an increased risk of aspiration. The risk of aspiration can be mitigated by thoughtful patient selection. Patients with altered mentation, active nausea/emesis, heavy secretion burden or recent gastroenteric interventions should not be started on NIV [23].

## 5. Challenging Clinical Situations Encountered During Liberation from NIPPV

### 5.1. Morbid Obesity

Morbid obesity, particularly in a central (abdominal) pattern, leads to increased airflow resistance, reduced functional residual capacity, abnormal respiratory mechanics, reduced respiratory muscle strength, atelectasis and reduced lung volumes [24]. These defects are associated with closure of peripheral lung units, abnormal ventilation to perfusion relationships, and hypoxemia. Many obese patients, especially those with BMIs over 40, have obstructive sleep apnea and obesity hypoventilation syndrome [25]. These patients may have underlying hypercapnia and chronic dependence on non-invasive ventilatory support. Cases are further complicated by comorbid conditions, including cardiac complications of obesity [26]. While there is a described phenomenon known as the obesity paradox, where higher body mass index is associated with improved outcomes in patients with hypoxic respiratory failure, research has failed to demonstrate this in specific patient populations, including COVID-19 cases [27]. When this underlying body physiology is coupled with acute insults such as pneumonia or aspiration, support of the patient with morbid obesity becomes increasingly challenging.

NIV is a proven modality in managing acute hypoxic respiratory failure in morbidly obese patients, including weaning from mechanical ventilation. Data shows a reduced risk of reintubation, increased ventilator-free days and a lower risk of nosocomial pneumonia in patients extubated to NIV [13]. While NIV is a proven modality for the management of acute respiratory failure in this population, it can be complex when a morbidly or super-morbidly obese patient with acute respiratory failure weans off non-invasive ventilatory support due to the high pressures needed to overcome thoracic impedance, prevent airway collapse and ensure airway compliance, oxygenation and ventilation in this patient population [28,29].

A carefully planned and protocolized weaning strategy, which focuses on slowly weaning pressure support and close monitoring, may increase the likelihood of successful weaning. Many of these patients may ultimately be discharged home with nocturnal NIV support [30]. A small study in France showed that 34% of patients treated for idiopathic acute respiratory failure who were morbidly obese and not on NIV at home at admission were discharged home with NIV at the end of their hospital stay [31]. Continuous mask ventilation in super-morbidly obese patients often comes with a lack of access to oral medication administration and the need for routing all therapies as intravenous formulations when necessary. The augmentation of PEEP by non-invasive ventilation offers distinct advantages over high-flow nasal cannula in obese patients, in addition to providing a mechanism for supporting ventilation and reducing in hypercarbia [13]. These facets of care for obese and super-obese patients with hypoxia make the coupling of non-invasive ventilation with continuous intravenous sedation agents such as dexmedetomidine ideal choices in the ICU.

### 5.2. Delirium and Dementia

A 2018 metanalysis found that 31–38% of ICU patients will suffer from delirium during their ICU stay [32]. Delirium is an important cause of prolonged NIV and NIV failure [33,34,35,36]. Mask intolerance leads to worsening agitation and is associated with an increased risk of delirium in patients started on NIV. Pre-existing cognitive impairment, dementia, older age and severity of illness are associated with higher rates of delirium in patients on NIV [34]. Delirium not only increases the risk of NIV failure, but it also interferes with weaning by impacting patient cooperation, limiting their ability to participate in weaning trials. Ensuring comfort through patient education and taking a collaborative approach to selecting a comfortable mask interface are imperative to ensure NIV success.

The management of delirium in the ICU is nuanced. The Society for Critical Care Medicine recommends multimodal, nonpharmacological interventions as the first-line treatment for delirium [37]. The MIND-USA trial did not reveal any difference in delirium-free days, ventilator-free days or mortality between critically ill patients with delirium treated with antipsychotics vs. placebo [35]. However, in patients with severe agitation in the setting of hyperactive delirium, a prevalent phenomenon in NIV complicated by delirium, antipsychotics can be used [36]. The loss of enteral access in these cases limits pharmacological options. While there is some signal that Seroquel may hasten the resolution of delirium, intravenous or intramuscular antipsychotics and dexmedetomidine are the mainstays of management of delirium in this patient population [38]. A 2024 metanalysis found that appropriate use of sedative and analgesic medications reduced the risk of endotracheal intubation and worsening delirium in patients on NIV. Dexmedetomidine was noted to be superior to other sedatives [39]. Benzodiazepines should be avoided due to the association with worsening delirium and prolonged ICU stays [40]. This weaning strategy is based on expert opinion as there is no direct data on weaning from NIV in delirium or dementia.

### 5.3. Advanced COPD

Advanced COPD presents unique challenges in initiation, management and weaning from non-invasive ventilation due to respiratory muscle weakness, persistent and rebound hypercapnia and dynamic hyperinflation [41,42]. In the critical care setting, NIV is not only the mainstay of management of acute hypoxic and hypercapnic respiratory failure in COPD, but it is also the modality of choice for weaning from invasive mechanical ventilation [3,4,12].

Patients with advanced COPD often have chronic hypercapnia due to a persistent ventilatory defect. When these patients are critically ill, it is important to differentiate acute hypercapnia from chronic hypercapnia and avoid over-ventilating these patients. Hypercapnia (PaCO2 > 45) with a normal PH (arterial PH > 7.35) and a high serum bicarbonate (>30 mEq/L) points towards a chronic respiratory acidosis with a concomitant compensatory metabolic alkalosis. Acute-on-chronic hypercapnia (as seen in COPD exacerbations) in this population is primarily managed with NIV. Early initiation of high-intensity NIV is associated with reduced rates of IMV and reduced length of ICU stay [43]. However, these patients require careful titration of minute ventilation and frequent blood gas monitoring to ensure a therapeutic range without causing hypocapnia, respiratory alkalosis and apnea.

Once clinical conditions improve, it is imperative to be strategic in weaning off NIV support. Patients with advanced COPD can suffer rapid deterioration and rebound hypercapnia on discontinuation of NIV and require careful, planned, slow weaning from support. The rapid shallow breathing index (RSBI) can be used to predict eligibility for weaning and predict successful weaning in these patients [44]. In the HAPPEN trial, IPAP was reduced by 1–2 cm H_2_O, ensuring tidal volume reduced by ≤5%, and heart rate and respiratory rate increased by ≤5%. An arterial blood gas was obtained two hours after every change in IPAP, and IPAP was not reduced by more than 4 cm of H_2_O in 24 h. Daytime NIV use was titrated down first, with patients resting on NIV, and NIV was discontinued once patients were on it for less than 6 h per day [43]. This represents a conservative approach to weaning in high-risk patients. However, there is some data that suggests that if patients tolerate 4 h of unassisted breathing without worsening of hypercapnia or respiratory distress, direct discontinuation of NIV is non-inferior to and associated with reduced ICU stay when compared to gradual weaning in COPD patients receiving nocturnal NIV support [45]. Using predictive tools like the RSBI can help tailor weaning strategies. There are gaps in understanding, as weaning strategies have not been studied head-on in patients with advanced COPD, and guidelines do not recommend a specific, universal weaning strategy [46]. However, in practice, in the high-risk patient population with COPD (baseline hypercapnia, nocturnal dependence on NIV, severe airflow obstruction, frequent exacerbations), protocol-directed, gradual weaning strategies reduce the duration of NIV and length of ICU stay by preventing rebound hypercapnia and prolonged respiratory failure [47].

### 5.4. Neuromuscular Disorders

Respiratory complications are a leading cause of morbidity and mortality in neuromuscular disorders. Respiratory muscle weakness leads to reduced lung volumes and compliance, causing a restrictive ventilatory defect and impaired cough, and bulbar muscle weakness increases aspiration risk [48]. These patients develop hypercapnia, nocturnal hypoxia and sleep-disordered breathing, often necessitating chronic NIV use. The use of non-invasive ventilation improves survival across the spectrum of neuromuscular disorders [49].

NIV is also the mainstay of management of acute respiratory failure in this patient population. In one study it was found that in patients with acute hypoxic respiratory failure in the setting of neuromuscular disease, NIV initiation successfully prevented IMV in 65% of the subjects started on NIV, and invasive mechanical ventilation was associated with increased risk of mortality [50]. Unlike the general population, these patients have static and progressive neuromuscular weakness that makes weaning from ventilatory support challenging [51]. The loss of enteral access while on NIV compounds weakness and malnutrition. Critical illness myopathy accelerates neuromuscular decline in these patients, leading to long-term NIV dependence. Conventional approaches to weaning may not be easily applicable in these patients, and there are distinct differences in the likelihood of weaning based on the specific underlying neuromuscular disease. Patients with advancing amyotrophic lateral sclerosis, for example, may embark on NIV support in home settings and, when they acutely decompensate and encounter ICU care, have low rates of weaning from NIV. Direct conversations with these patients and their caregivers on goals of care and expectations of ICU outcomes are best carried out early in ICU admissions to avoid prolonged NIV support and early tracheostomy where this is a desired goal on the part of the patient. Conversely, patients with reversible neuromuscular disorders or those with modifiable weakness, such as Guillain–Barre syndrome may be successfully supported by NIV and strategically liberated from this modality as their underlying disorder responds to immune modulating therapies.

The most important aspects of supporting patients with neuromuscular disorders with NIV in inpatient settings lie in determining early if the driving component of respiratory failure is a progression of an incurable disease or an inflection point related to a reversible secondary medical condition. Rapidly identifying secondary causes of respiratory failure in these heterogeneous patients is paramount to successful weaning from NIV and overall survival to discharge. Early engagement with palliative care should be commonly included in the treatment paradigm of patients with progressive neuromuscular diseases being managed in ICU settings with NIV to avoid unwanted therapies and complications.

### 5.5. Pulmonary Hypertension

Patients with pulmonary hypertension (PH) have variable rates of progression and development of hypoxic respiratory failure, depending on the etiology and group of PH.

Group 1 patients (pulmonary arterial hypertension or PAH) comprise a much smaller cohort comparatively and have a more progressive course. They are often managed with non-invasive mechanical ventilation in the acute setting with the delivery of inhaled PAH therapies as mean pulmonary artery pressures worsen [52,53]. Additionally, patients with sympathetic crashing acute pulmonary edema (SCAPE) can present in extremis and have been successfully managed with emergent non-invasive positive-pressure ventilation coupled with intravenous nitroglycerin and diuresis [54]. Therapeutic delivery of inhaled vasodilator therapies has been and can be achieved through non-invasive ventilation support devices. While this approach has been growing in clinical practice globally, clinical trials demonstrating efficacy and standardized approaches to implementation are lacking [55].

Patients with pulmonary hypertension secondary to group 3 pulmonary hypertension, i.e., PH associated with primary lung disease and hypoventilation, demonstrate significant improvement with NIV, including a reduction in mean pulmonary artery pressure by approximately 18 mmHg and improved exercise capacity [56]. Similarly, in group 2 PH secondary to left heart disease, NIV is beneficial in the management of cardiogenic pulmonary edema.

Positive-pressure ventilation increases intrathoracic pressure, reducing venous return to the heart. The mean pulmonary artery pressure also reduces significantly because of NIPPV therapy, improving right ventricular pressure and volume overload in patients with pulmonary hypertension [57,58]. The American Heart Association (AHA) recommends NIV initiation post-extubation in patients with PH to prevent worsening right heart failure, pulmonary hypertension crisis and reintubation [59]. In patients with pulmonary hypertension, NIV use is associated with a lower risk of intubation compared to high-flow nasal cannula [60]. As a result, NIV is a common treatment modality for the management of acute respiratory failure in patients with pulmonary hypertension. However, once the acute illness is over, transitioning from NIV to spontaneous breathing can be challenging.

The discontinuation of NIV leads to a decrease in intrathoracic pressure, an increase in preload, increase in pulmonary vascular resistance and pulmonary arterial pressure and an increase in left and right ventricular afterload [61]. In addition, the metabolic stress of spontaneous work of breathing may be overwhelming in these cases, leading to failure to wean from NIV support. Patients with substantial PH and hypoxic respiratory failure supported concomitantly with inhaled epoprostenol or nitric oxide therapy benefit from switching to longer acting oral therapies or intravenous vasodilator therapies, as support from NIV becomes sporadic, to bridge continuous management of pulmonary vasoconstriction. Improvements in hemodynamics and hypoxia in this patient population are best coupled with plans for transitioning PH therapy rather than abrupt cessation and risk of rebound hypoxia. In conclusion, slow and strategic weaning is necessary to ensure success of liberation from NIV in patients with pulmonary hypertension.

Evidence addressing ideal weaning strategies in pulmonary hypertension is extremely limited. As stated, prior studies have focused on the role of NIV in the management of acute hypoxic respiratory failure in this patient population and its role in facilitating inhaled vasodilator therapy. Limited attention has been given to optimal strategies to transition off NIV. In the clinical setting, this means that standard weaning protocols may not account for the unique hemodynamic instability associated with positive-pressure ventilation in right ventricular failure. Prospective studies comparing structured weaning protocols (rapid vs. gradual weaning and stepwise pressure reduction strategy), as well as studies of hemodynamic impact of NIV withdrawal and mitigation strategies, are imperative for establishing evidence-based protocols for the management of this high-risk population.

## 6. Approach to Liberation in Complex Patients

Utilization of NIV support in patients presenting to emergency care and intensive care units has evolved from use in highly selected patient populations to widespread deployment in medical and surgical intensive care and post-operative settings. Like any advancement in medical care with broad applications, NIV is at times extended beyond its realm of utility and overutilized in clinical settings where intubation and conventional mechanical ventilation would offer better outcomes for patients [62].

Incorporating specialized patient populations who may benefit from NIV support during inpatient care into a larger algorithm of consideration presents a comprehensive approach to deployment of this support. We propose a nuanced approach to liberation from NIV using dichotomous and progressive decision steps that aid in understanding the complexity of a patient’s underlying medical conditions, guide the clinician in modifying the goals of NIV and align the outcomes closer with patient expectations and improve their overall comfort.

### 6.1. Assessing Readiness to Wean from Non-Invasive Ventilatory Support

**Figure 3:**
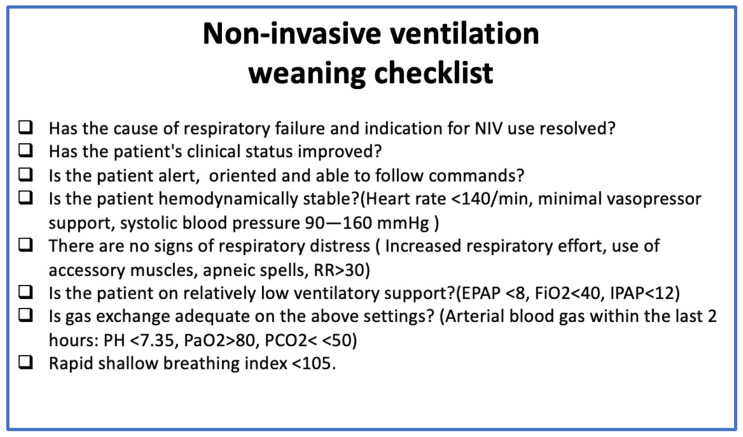
Checklist to assess readiness to wean from non-invasive ventilation.

There is no standardized approach to determine readiness for weaning or predict the success of weaning. Weaning strategies vary not only from center to center but also among providers within the same facility. There is no standardized approach to weaning from NIV. However, there are some universal criteria that aid in assessing readiness to wean from non-invasive ventilatory support regardless of disease process (Figure 3). These criteria are adapted from expert opinion and data extrapolated from weaning trials in invasive mechanical ventilation. For example, in patients with COPD, the rapid shallow breathing index (RSBI) is shown to accurately predict the success of weaning from NIV [48]. While direct data for other complex situations described in our review does not exist, given the evidence of success in weaning from IMV and NIV, the RSBI can be applied to most cases of respiratory failure requiring NIV [63]. If patients do not meet these criteria for weaning, subsequent weaning efforts are likely to fail.

### 6.2. Special Considerations

- In patients with chronic hypercapnia PaCO_2_ > 50 is acceptable if PH remains in the normal range (7.35–7.45).
- In patients with neuromuscular disease, the RSBI may not be particularly helpful. In these patients the diaphragm thickening fraction (DTF) can be used as an additional tool to predict successful weaning from NIV. DTF is an ultrasound-based measurement of diaphragmatic strength. Diaphragm thickness is sonographically measured at end-expiration and end-inspiration. The percentage change in diaphragmatic thickness (DTF) accurately predicts weaning success [64].
- In patients with pulmonary hypertension requiring inhaled vasodilator therapy in the ICU, any ventilator weaning strategy must incorporate the assessment of continued need for vasodilator therapy. Weaning from NIV must proceed concomitantly with a transition to an oral/intravenous domiciliary regimen.
- Agitation and delirium are independent risk factors for failure to wean from NIV. Agitated patients may need initiation and continuation of antipsychotic (Seroquel, haloperidol) or sedative (dexmedetomidine) medications.

### 6.3. Weaning Strategies

Given the complexity of the patient population described in our review, once the primary assessment for weaning is completed, weaning strategies must be individualized not only to the patient but also to the disease process.

### 6.4. Gradual Weaning vs. Sudden Discontinuation

**Figure 4:**
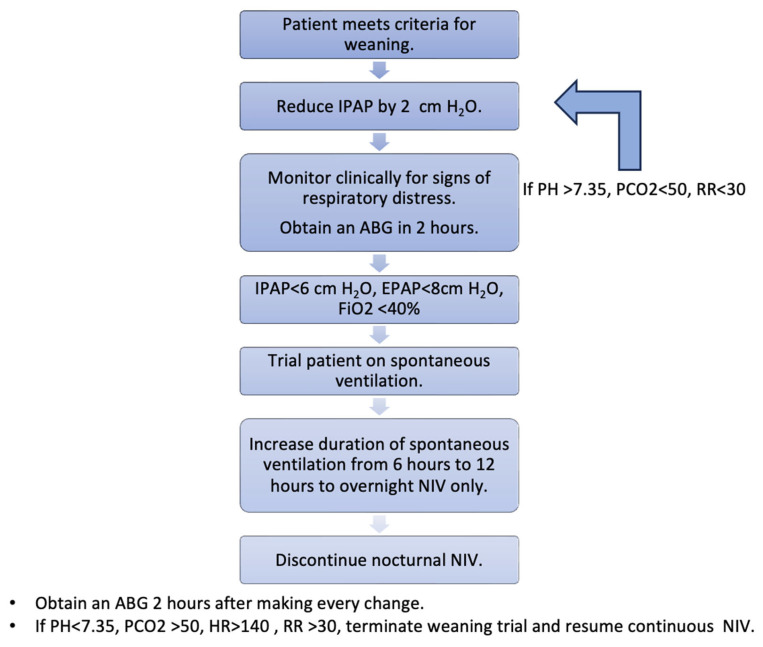
Suggested protocol for gradual weaning from NIV.

Patients can be gradually weaned off ventilatory support or abruptly taken off NIV depending on disease process and baseline physiology. A slow weaning strategy has shown good outcomes with reduced risks of weaning failure in patients with COPD [65]. This can be extrapolated to other complex disease processes. Particularly in patients with chronic hypercapnic respiratory failure, domiciliary use of NIV and prolonged ICU stay with neuromuscular weakness, a slow weaning strategy is preferred. DTF can be used in addition to the above weaning assessment to identify patients that may benefit from a gradual, prolonged NIV weaning strategy. Figure 4 outlines our proposed weaning strategy.

On the other hand, abrupt discontinuation is appropriate in patients with acute respiratory failure without risk factors for relapse off NIV. For instance, NIV was successfully discontinued in patients with severe COPD requiring NIV for acute respiratory failure, who tolerated 4 h of unassisted breathing, without a prolonged nocturnal NIV wean [49]. However, notably, these patients were not on domiciliary NIV support.

### 6.5. Disease Specific Considerations

As outlined in our review, the specific disease states discussed present distinct pathophysiologic mechanisms that warrant an individualized approach to weaning. Table 1 outlines disease-specific concerns and strategies to optimize weaning.

## 7. Summary

In the last two decades, non-invasive positive-pressure ventilation has evolved from a specialized intervention used in specific cases of respiratory failure to a mainstay of respiratory support in critically ill patients. While NIV has become ubiquitous in the ICU setting, guidelines, protocols, universal assessment tools, predictive scores and comparative studies to guide the initiation, management and weaning of NIV are lacking. NIV weaning is physician-driven, based on clinical experience, practice style and preference. There are specific high-risk patient populations that require a careful and nuanced approach to ventilator weaning. We describe patients with advanced COPD or obesity with baseline hypercapnia and domiciliary NIV dependence, patients with neuromuscular disease and progressive respiratory failure, patients with PH requiring concomitant NIV and inhaled vasodilator therapy and patients with delirium unable to co-operate with the face mask interface and participate in weaning protocols. Each one of these unique patient populations requires a tailored approach to weaning. We provide a universal weaning checklist for high-risk populations. We then describe nuances to weaning in each unique patient population and special considerations. Finally, we recommend a gradual weaning strategy for these patients. A protocol for gradual weaning is provided in our paper. Table 2 summarizes key findings of the evidence cited in our review.

## Figures and Tables

**Table 1:** Disease specific weaning considerations.

Clinical Condition	Key Pathophysiology	Weaning Strategies	Causes of Weaning Failure	Clinical Considerations
**Morbid Obesity**	Low chest wall compliance, sleep-disordered breathing, upper airway obstruction, reduced respiratory muscle strength.	Daytime liberation with continued nocturnal NIV. Gradually wean pressure support to identify optimal settings.	Airway obstruction, nocturnal hypercapnia, daytime somnolence and hypoventilation and airway obstruction.	Monitor arterial blood gases in the morning. If nocturnal hypercapnia or hypoxemia persists, consider domiciliary positive airway pressure (PAP) therapy.
**Delirium and dementia**	Mask intolerance, agitation and anxiety, asynchrony and inability to follow commands and cooperate.	Patient education, caregiver involvement, supervised weaning, non-pharmacological interventions for delirium and, as a last resort, antipsychotics and dexmedetomidine may be used.	Mask refusal, poor seal, asynchrony, agitation and distress, inability to cooperate and aspiration events.	NIV should be discontinued rapidly where able. When gradual weaning is needed, it must be supervised, with minimal sedation used to ensure tolerability.
**Advanced COPD**	Persistent ventilatory defect, dynamic hyperinflation, persistent hypercapnia and respiratory muscle weakness.	RSBI to predict eligibility for weaning. Individualize prolonged vs. rapid weaning strategy. In high-risk patients gradually wean daytime duration followed by nocturnal weaning.	Recurrent hypercapnia, respiratory muscle fatigue, sleep-related hypoventilation and mucus clearance.	Monitor for rebound hypercapnia and nocturnal hypercapnia. Patients with persistent nocturnal hypercapnia and daytime somnolence benefit from domiciliary biPAP initiation.
**Neuromuscular disorders**	Neuromuscular weakness, nocturnal hypoventilation and ineffective cough and mucus clearance.	Initiate airway clearance protocols, slowly reduce pressure support, individualize weaning to disease process and gradually wean nocturnal support.	Atelectasis, mucus plugging, recurrent/persistent hypercapnia and fatigue.	RSBI is unreliable. Use DTF to determine readiness for weaning. Patients should be monitored for nocturnal hypercapnia and hypoxemia. High likelihood of domiciliary NIV needs.
**Pulmonary hypertension**	Reduced RV preload with positive-pressure ventilation, sensitivity to acute changes in intrathoracic pressure and increased pulmonary vascular resistance from hypoxemia.	Individualize to group of PH. NIV weaning must occur concurrently with transition from inhaled to systemic vasodilator therapy. Use lowest effective airway pressure, avoid abrupt changes in pressures and wean pressure support gradually.	Hypotension, worsening RV failure and severe hypoxemia.	Weaning should be gradual and very closely monitored. Hypoxia should be avoided given the risk of pulmonary vasoconstriction and hemodynamic instability. Group 3 patients may need and benefit from domiciliary nocturnal NIV support.

**Table 2:** Trials directly studying non-invasive ventilation in unique patient populations, key findings and clinical recommendations.

Clinical Condition	Study	Key Finding	Clinical Recommendations
**Morbid obesity**	Bry et al. (2018) [31] (retrospective study)	64% of patients with morbid obesity and acute hypoxic respiratory failure requiring BiPAP were discharged home with NIV. These patients had a higher BMI and required higher initial IPAP settings.	Domiciliary NIV should be strongly considered in morbidly obese patients following acute respiratory failure.
**Delirium**	Zhang et al. (2021) [34] (prospective study)	18% of patients on NIV developed delirium. Delirium was strongly associated with NIV failure (37.8% in the NIV group vs. 21% in non-NIV group), ICU mortality (33.2% vs. 14.3%) and hospital mortality (37.2% vs. 17%).	Patients on NIV should be screened for delirium daily, and early non-pharmacological and, if needed, pharmacological interventions should be implemented.
**Delirium**	Yang et al. (2023) [39] (systematic review and meta-analysis)	Sedation/analgesia, compared to no sedation, and dexmedetomidine, compared to other sedatives, were associated with reduced rates of endotracheal intubation, delirium incidence, duration of NIV and ICU length of stay, with no significant difference in all-cause mortality.	Consider early initiation of sedation and analgesia in patients with delirium on NIV after optimizing non-pharmacological risk factors. Dexmedetomidine should be the agent of choice when not contraindicated.
**COPD**	Yu et al. (2021) [44] (retrospective study)	65.9% of the 85 enrolled patients were successfully weaned from NIV. The RSBI was the strongest predictor of weaning success with an area under the curve of 0.804 (*p* < 0.001).	The RSBI should be measured when selecting patients suitable for weaning from NIV. A high RSBI should prompt a gradual weaning strategy.
**COPD**	Sellares et al. (2017) [45] (randomized controlled trial)	Prolonged nocturnal weaning in patients with COPD, on NIV for acute hypercapnic respiratory failure, does not prevent relapse and results in longer ICU stays when compared to direct discontinuation if patients tolerate being off NIV continuously for 4 h.	NIV should be directly discontinued in patients who can tolerate at least 4 h of unassisted breathing.
**COPD**	Duan et al. (2012) [47] (randomized controlled trial)	Protocol-directed weaning from NIV in patients with acute exacerbation of COPD resulted in reduced median duration of weaning from 5 to 3 days (*p* < 0.001) and reduced ICU length of stay from 9 to 7 days (*p* = 0.002) when compared to physician-directed weaning.	Weaning from NIV in COPD should be protocol-directed rather than physician-directed.
**Pulmonary hypertension**	Held et al. (2013) [56] (retrospective study)	In patients with severe pulmonary hypertension secondary to hypoventilation, 3 months of NIV was associated with a significant reduction in mean pulmonary artery pressure (18 mmHg), reduced proBNP levels, improved 6 min walk distance and increased work rate.	NIV should be considered standard therapy for patients with group 3 PH. Patients started on NIV in the setting of acute respiratory failure should be assessed for long-term NIV needs.
**Neuromuscular disease**	Poddighe et al. (2024) [64] (systematic review and comparative meta-analysis)	Diaphragmatic thickening fraction (DTF) and diaphragmatic excursion (DE) demonstrate the highest accuracy in predicting weaning success from mechanical ventilation in patients with neuromuscular disease.	Instead of the RSBI, DTF and DE assessments should be used to predict weaning success from NIV in patients with neuromuscular disease.

## Data Availability

No new data were created or analyzed in this study.

## Abbreviations

The following abbreviations are used in this manuscript:

NIV	Non-invasive ventilation
PEEP	Positive end-expiratory pressure
IMV	Invasive mechanical ventilation
BiPAP	Bilevel positive airway pressure
CPAP	Continuous positive airway pressure
COPD	Chronic obstructive pulmonary disease
CHF	Congestive heart failure
COVID-19	Coronavirus disease 2019
ICU	Intensive care unit
NIPPV	Non-invasive positive-pressure ventilation
EPAP	End-expiratory positive airway pressure
IPAP	Inspiratory positive airway pressure
RSBI	Rapid shallow breathing index
PH	Pulmonary hypertension
PAH	Pulmonary arterial hypertension
SCAPE	Sympathetic crashing acute pulmonary edema
AHA	American Heart Association
DTF	Diaphragm thickening fraction
